# Experimental study on damage characteristics of Beishan granite under single loading and multiple loading with AE techniques

**DOI:** 10.1038/s41598-023-35921-y

**Published:** 2023-05-30

**Authors:** Zihui Wang, Guanglei Zhou, Xinbo Ge

**Affiliations:** 1 State Key Laboratory of Mining Disaster Prevention and Control Cofounded By Shandong Province and the Ministry of Science and Technology, Shandong University of Science and Technology, Qingdao, 266590 Shandong People’s Republic of China; 2 College of Energy and Mining Engineering, Shandong University of Science and Technology, Qingdao, 266590 Shandong People’s Republic of China

**Keywords:** Civil engineering, Mechanical engineering

## Abstract

An insight into the damage characteristics of host rock is important for permanent disposal of high-level radioactive waste (HLW). Single loading and multiple loading tests for Beishan granite were carried out. The acoustic emission (AE) monitoring system was utilized simultaneously to measure the damage evolution in the specimens. The results show that, during single loading the AE signal has been maintained at a very high level after dilatancy. But a decreasing trend of AE signals can be observed when the axial stress approaches the peak stress. While during multiple loading, AE signals are mainly generated in the loading period in the elastic stage. However, in the failure and residual stages, AE signals generated in the unloading period are considerable. Most of the AE waveforms have a dominant frequency of less than 200 kHz and amplitude of less than 80 dB, and three frequency bands are obtained. In addition, the amplitude increases with the increase of loading stress. Furthermore, the obtained AE *b-value*s indicate that the relative proportion of micro- to macro-cracks increases first and then decreases with increasing confining pressure. And the AE *a*-values and *b-*values confirms that micro cracks develop rapidly in the damage stage and lead to the formation of macro cracks in the residual stage for the specimens experienced multiple loading.

## Introduction

Granite, as a host rock of good stability, high strength and low permeability, is considered to be an ideal engineering surrounding rock for high-level radioactive waste (HLW) disposal. Now China is building HLW repository in Beishan of Gansu Province, with granite chosen as surrounding rock^1,2^. Therefore, a better understanding of the progressive failure characteristics of Beishan granite plays an important role in the safety of the repository.

It is well known that the propagation of micro cracks is responsible for the rock failure^3,4^. Generally, the micro cracks develop along with the axial loading direction^5^. And the crystal boundary is the preferred channel for the development of micro cracks^6^. When the stress reaches a certain level, the open crack will lead to rock dilatancy^7–9^. And many scholars have marked several characteristic stresses according to the acoustic emission (AE) characteristics of crack development, namely crack closure stress, crack initial stress and crack damage stress^10,11^. Furthermore, damage process of rock materials has been widely investigated by analyzing AE parameters^12–14^. And AE signals with varying amplitudes can be interpreted to reveal different types of cracks^15,16^.

For rock-like materials, the strength and deformation characteristics can be obtained by one-time loading failure experiment. However, in practical engineering, most rock materials are damaged by repeated loading. Therefore, it is very important to study the failure characteristics and cumulative damage of rock materials under different stress states. And loading–unloading test simulating excavation provides an effective way to study the damage and deformation of rocks. In general, the elastic and plastic strain components of rock during deformation can be distinguished through the loading–unloading experiments^17^. And the influence of accumulative damage on crack initial stress, crack damage stress and peak stress can also be investigated^18,19^. The input energy, elastic energy and dissipated energy in each cycle can be calculated to analyze rock failure process from the perspective of energy^20,21^. Furthermore, Xiao et al. evaluated the advantages and disadvantages of the 6 damage variables according to uniaxial loading–unloading experiments of granite^22^. Moreover, Qiu et al. designed an incremental loading–unloading test for quantitative analysis of the pre peak damage behavior of marble in Jinping^23^. In a word, cyclic loading test is an important method to investigate rock failure process.

In the present study, conventional triaxial compression (CTC) test is carried out to obtain basic mechanical properties of Beishan granite. And AE parameters are analyzed to reveal the crack evolution. Then cyclic loading test is conducted to study the influence of cumulative damage on crack development. And the changes of AE frequency and amplitude at each stage, as well as the overall ratio of micro-cracks to macro-cracks, are analyzed.

## Laboratory test settings

### Preparation of rock specimens

Granite blocks were taken from the Beishan area, Gansu Province, China. Beishan granite is a medium and fine-grained granite with grain sizes ranging from 0.25 to 4.4 mm. Cylindrical granite specimens with diameter of 50 mm and length of 100 mm were prepared. The specific information of each specimen is listed in Table 1.

**Table 1:** Sample information.

No	Samples	Diameter/mm	Length/mm	Mass/g	Density(g/cm^3^)	Confining pressure/MPa
1	CTC-1	50.04	100.15	513.52	2.607	0.5
2	CTC-2	50.00	100.11	513.93	2.615	2
3	CTC-3	49.93	100.02	511.50	2.612	5
4	CTC-4	50.04	99.87	512.17	2.608	10
5	CTC-5	50.12	100.26	516.30	2.610	15
6	CTC-6	50.05	100.24	514.34	2.608	30
7	CTC-7	49.88	100.18	510.22	2.606	0.5
8	CTC-8	49.87	100.04	510.65	2.613	2
9	CTC-9	49.84	99.92	509.58	2.614	5
10	CTC-10	49.97	99.98	511.27	2.608	10
11	CTC-11	50.19	100.22	516.60	2.605	15
12	CTC-12	50.12	100.28	516.00	2.608	30
13	CYC-1	49.93	100.18	511.81	2.609	0.5
14	CYC-2	50.02	100.25	512.68	2.602	2
15	CYC-3	49.96	100.24	511.56	2.603	5
16	CYC-4	50.10	99.99	514.61	2.611	10
17	CYC-5	49.92	100.06	511.81	2.613	15
18	CYC-6	49.66	99.97	506.84	2.618	30

*CTC-no.* represents specimen number in conventional triaxial compression (CTC) test, *CYC-no.* represents specimen number in cyclic loading–unloading (CYC) test.

### Mechanical test facility and testing procedure

**Figure 1:**
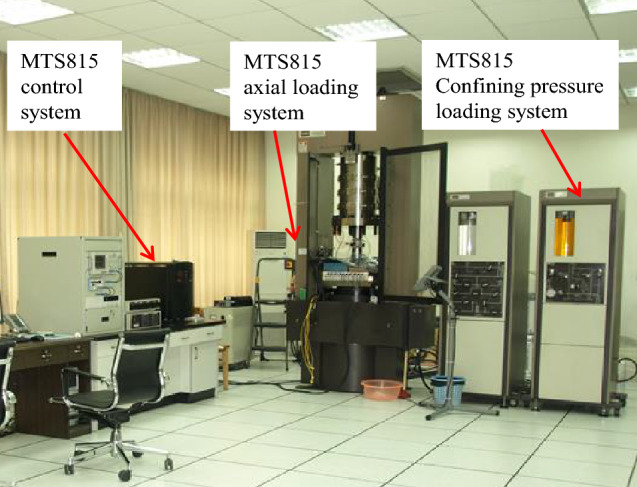
MTS815 Flex Test GT rock mechanical test machine.

**Figure 2:**
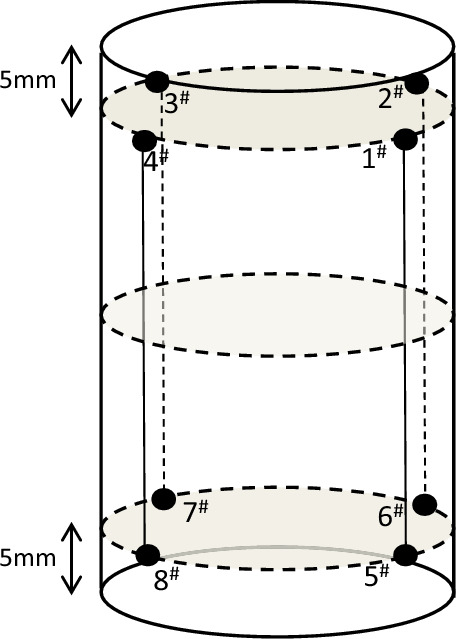
Layout of AE sensors.

All the tests were carried out with a MTS815 Flex Test GT rock mechanical test machine, as shown in Fig. 1. A 12-channel portable AE monitoring facility was utilized to record AE activity during the tests. Superior low noise and low threshold performance have been achieved with this AE system, with up to 40 MHz sample acquisition and real time sample collecting. During the tests, the trigger threshold of AE was set to 28 dB. Eight AE sensors were used in the tests. And four of them were a group and distributed evenly in a plane 5 mm away from the boundary of the specimen outside on the test machine pressure cell, as shown in Fig. 2.

Twelve specimens, set as 2 groups, were used for CTC test. The confining pressures are set to 0.5, 2, 5, 10, 15 and 30 MPa, respectively. In the first step, a vertical load of about 3 kN is applied in order to fix the position of the specimen. Then, the desired confining pressure is reached with a constant loading rate of 0.05 MPa/s to ensure that the specimen is under uniform hydrostatic stresses. Afterwards, the axial stress is increased with a constant loading rate of 500 N/s. Lateral deformation control was used when the axial stress approaches about 60% of the peak stress.

Six specimens, set as 1 group, were used for triaxial cyclic loading–unloading (CYC) test. The confining pressures are set to 0.5, 2, 5, 10, 15 and 30 MPa, respectively. The designed maximum stresses in each cycles are listed in Table 2. In the sixth cycle, the axial stress is loaded to the peak stress of the specimen. In the cycles after the peak stress, the peak stress of each cycle is determined according to the actual loading condition. Other loading steps are consistent with CTC test.

**Table 2:** The designed maximum stresses in each cycles.

Confining pressure/MPa	1st cycle/MPa	2nd cycle/MPa	3rd cycle/MPa	4th cycle/MPa	5th cycle/MPa
0.5	20	40	60	80	90
2	25	50	75	100	112.5
5	30	60	90	120	135
10	40	80	120	160	180
15	50	100	150	200	225
30	70	140	210	280	315

## Experimental results

### CTC tests

#### Stress strain curves

**Figure 3:**
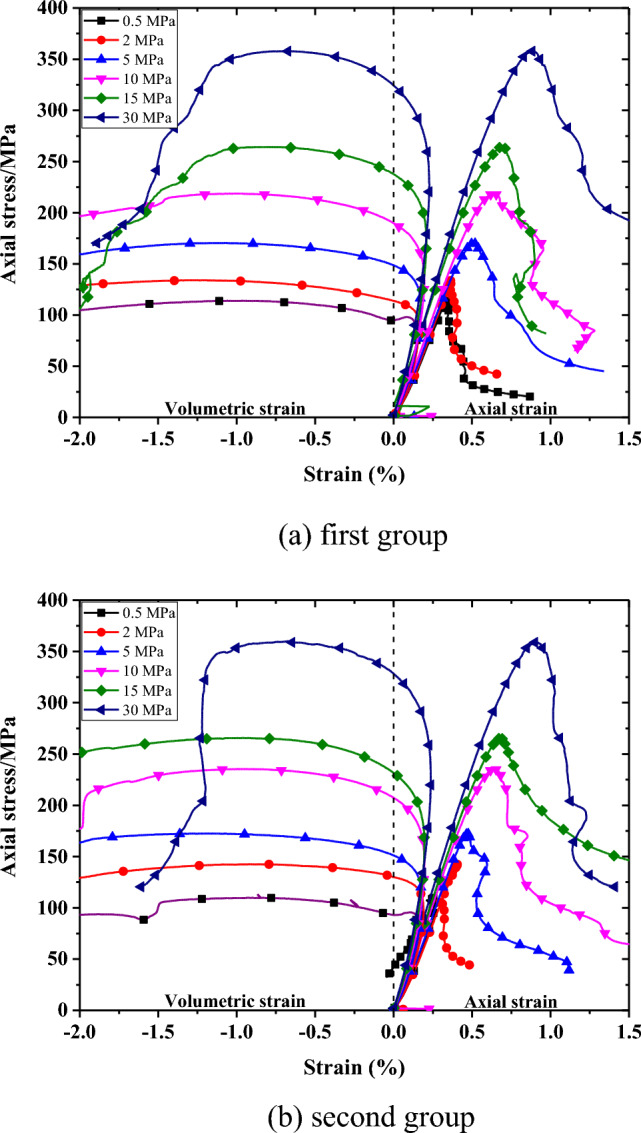
Stress–strain curves of Beishan granite under different confining pressures during CTC tests.

Typical stress–strain curves for Beishan granite under CTC tests are illustrated in Fig. 3. At the initial loading period, the non-linear behavior can be ignored. And the axial stress increases linearly with axial strain. The stress–strain curves begin to present non-linear behavior when the axial stress exceeds about 60% of the peak stress. After peak stress, the axial stress decreases to approach a relatively stable residual stress.

Table 3 lists the characteristic stresses and strain of Beishan granite in CTC tests. Under relatively low confining pressure, the ratio between σ_d_ and σ_c_ is relatively high, which is opposite to the ratio under relatively high confining pressure. ε_d_ and ε_c_ increase with increasing confining pressure. But their ratio shows decreasing trend with increasing confining pressure.

**Table 3:** Strength and strain parameters of Beishan granite under different confining pressures during CTC tests.

Group	Confining pressure/MPa	Peak		Dilatancy		ε_d/_ε_c_	σ_d_/σ_c_
		ε_c_	σ_c_	ε_d_	σ_d_		
1	0.5	0.344	113.99	0.240	79.26	0.698	0.695
	2	0.362	134.00	0.249	86.93	0.688	0.649
	5	0.507	170.45	0.299	110.21	0.590	0.647
	10	0.636	218.80	0.330	133.01	0.519	0.608
	15	0.689	264.26	0.379	175.30	0.550	0.663
	30	0.882	357.82	0.468	228.35	0.531	0.638
2	0.5	0.252	109.78	0.231	79.31	0.917	0.722
	2	0.408	142.50	0.280	96.21	0.686	0.675
	5	0.471	172.47	0.286	108.3	0.607	0.628
	10	0.644	235.45	0.340	143.59	0.528	0.610
	15	0.695	266.07	0.366	162.56	0.527	0.611
	30	0.904	360.15	0.468	222.29	0.518	0.617

ε_c_ presents the axial strain at peak stress, σ_c_ present the peak stress, ε_d_ presents the axial strain at dilatancy stress, σ_d_ present the dilatancy stress.

#### AE hits and AE events

**Figure 4:**
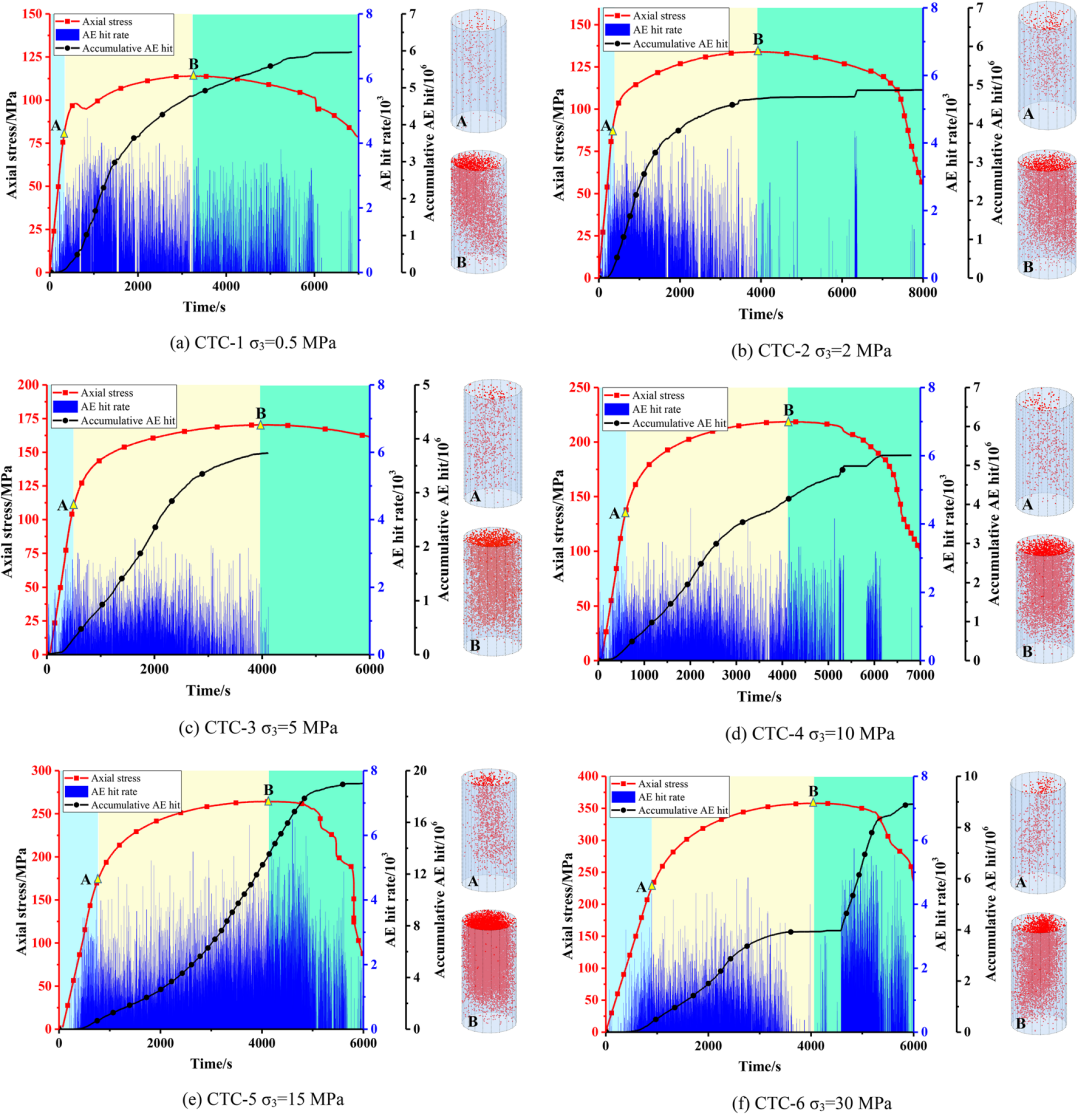
AE characteristics of Beishan granite under different confining pressures during CTC tests.

Figure 4 presents the evolution of the AE hits and AE events for granite specimens subjected to different confining pressures. Point A and B presents dilatancy and peak stress points, respectively.

We can see that the AE hit rate exhibits increasing trend before dilatancy. And AE events begin to gather on the pre macro-fracture surface. But after dilatancy, it illustrates different development trends. Under the confining pressure 0.5 and 2 MPa, the AE hit rate continues to increase for a short time after dilatancy, and then maintains a downward trend before peak stress. Under the confining pressure 5, 10 and 30 MPa, the AE hit rate is relatively stable after dilatancy. And when the axial stress approaches the peak stress, the AE hit rate decreases. Under the confining pressure 15 MPa, the AE hit rate is in a state of increasing trend between dilatancy and peak. It is worth noting that the decreasing of AE count rate is observed when the axial stress approaches the peak stress in some tests. In general, the AE count rate increases with the increasing stress. Because high stress can cause many new cracks. However, the generation of numerous cracks may lead to the formation of macro cracks or shear plane^16^, which can cut off the AE wave. And the AE signals cannot be received by AE sensors. As a result, the AE count rate may show decreasing trend when the rock approaches failure.

#### AE amplitude and frequency

**Figure 5:**
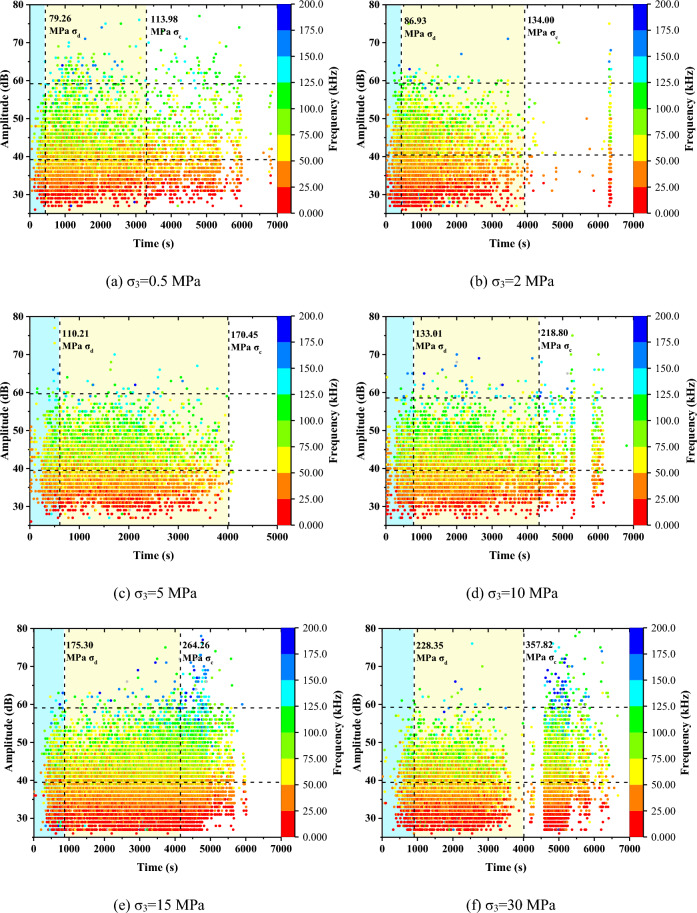
AE amplitude and frequency of Beishan granite under different confining pressures during CTC tests.

Figure 5 presents the dominant frequency-amplitude distributions of Beishan granite specimens under different confining pressures. As shown in Fig. 5, most of the AE waveforms have a dominant frequency of less than 200 kHz and amplitude of less than 80 dB. And High amplitude is usually accompanied by high frequency.

Compared to relatively low confining pressures, higher confining pressure often lead to generate more AE signals with higher amplitudes. Most AE signals of high amplitude, higher than 60 dB, are detected during the post-peak stage under relatively high confining pressures, indicating that violent cracking and fracturing occur in the granite specimen.

No matter in the loading stage or residual stage, AE signals can be divided into three frequency bands. The low-frequency band is in the range of 0–50 kHz, the intermediate frequency band is in the range of 50–125 kHz, and the high-frequency band is in the range of 125–200 kHz. Before dilatancy, almost all signals cluster within the low- and intermediate-frequency bands. Between the dilatancy point and the peak stress, although signals within the high-frequency band begin to occur, their number is relatively small. Only in the post peak stage of high confining pressure can the high-frequency signal have a certain scale.

#### AE *b*-value

In terms of the AE technique, *b-*value is often used to study the damage process. And it is calculated by the following formula^24^:

$$ \log_{10} \left( {\text{N}} \right) = a - b\left( {\frac{{A_{{{\text{dB}}}} }}{20}} \right) $$

where A_dB_ is the amplitude of AE in dB and N is the number of AE hits with amplitudes greater than A_dB_. The parameter *b-*value represents the ratio of small to large events, and the *a-*value is a measure of the level of AE hits^25^.

**Figure 6:**
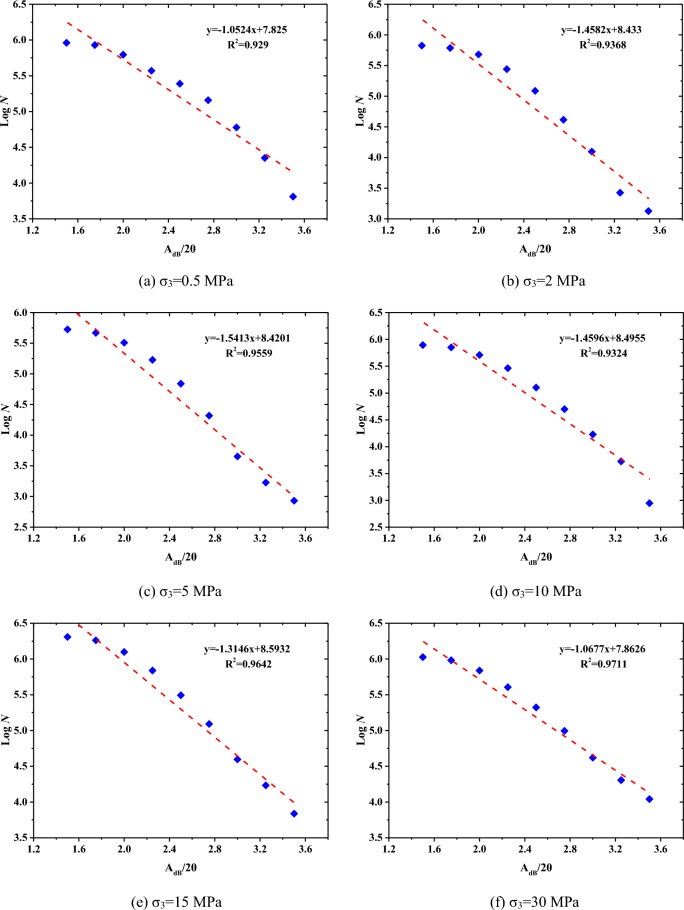
Calculation of b-value in CTC tests.

Figure 6 shows the linear fitting to calculated *a*-values and *b-*values for Beishan granite specimens exposed to different confining pressures during the whole loading process. The fitting results indicate that the *b-*values, i.e., the slopes of the fitting lines, are 1.0524, 1.4582, 1.5413, 1.4596, 1.3146 and 1.0677. The *b-value* increases first and then decreases with increasing confining pressure. This indicates that the relative proportion of micro- to macro-cracks increases first and then decreases with increasing confining pressure. Under relatively low confining pressure, it is easy to generate macro-cracks. However, under relatively high confining pressure, due to the strong confinement, the rock specimen needs to generate more microscopic cracks before it can be destroyed. Therefore, in both cases the *b-*value is lower.

The *a-value*s for granite specimens under 0.5, 2, 5, 10, 15 and 30 MPa are confining pressures 7.825, 8.433, 8.4201, 8.4955, 8.5932 and 7.8626, respectively. The *a-*value shows increasing trend with increasing confining pressure. This indicates that the numbers of AE hits detected from specimens increase with increasing confining pressure.

### CYC test

#### Stress strain curves

**Figure 7:**
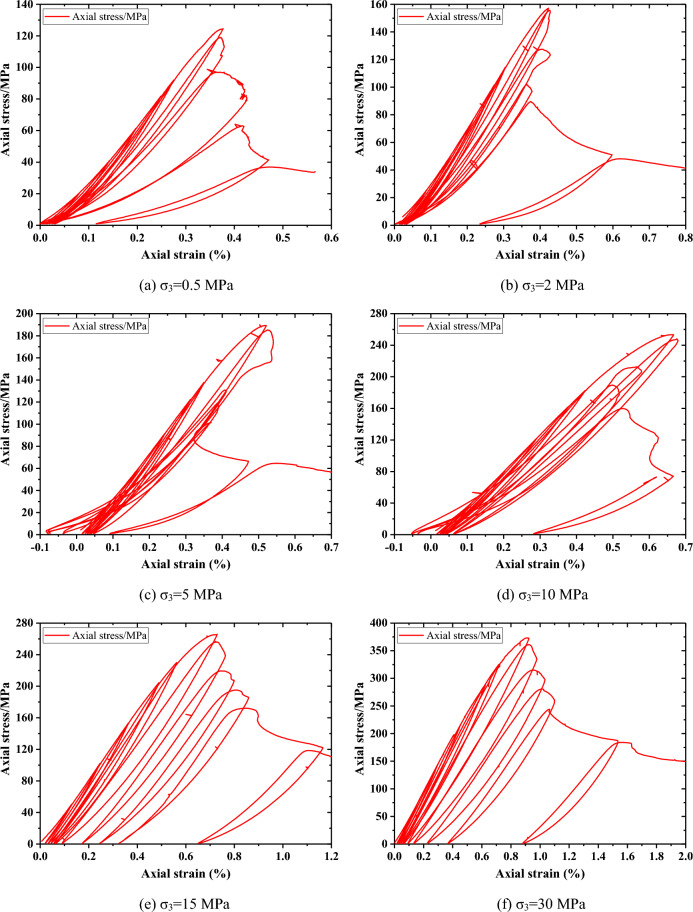
Stress–strain curves of Beishan granite under different confining pressures during CYC tests.

The stress strain curves in CYC tests are shown in Fig. 7. It should be noted that some strain data are biased due to the difficulty in controlling the experimental machine after the peak stress. The maximum axial stresses in each cycle are shown in Table 4. It can be seen that the maximum stress of each cycle before the peak is basically consistent with the set stress, which ensures the reliability of the experiment.

**Table 4:** Peak stress in each cycle under different confining pressures during CYC tests (MPa).

Confining pressure	1	2	3	4	5	6(P)	7	8	9	10	11
0.5 MPa	20.48	40.91	61.34	81.76	91.99	124.59	119.03	97.05	64.01		
2 MPa	25.50	50.95	76.38	101.82	114.55	157.11	155.72	127.44	101.85	89.65	48.14
5 MPa	30.66	61.27	91.87	122.49	137.79	189.30	185.37				
10 MPa	40.64	81.20	121.79	162.37	182.68	253.50	248.10	212.67	189.53	159.93	73.15
15 MPa	51.13	102.25	153.34	204.44	229.98	266.00	256.84	219.45	195.24	172.20	118.60
30 MPa	72.34	144.62	216.98	289.23	325.32	373.25	361.05	315.09	280.76	243.95	184.06

P indicates peak stress.

In the initial loading stage, the stress strain curves show non-linear behavior. However, as the confining pressure increases, this phenomenon becomes weaker and weaker. The relatively high confining pressure leads to the closure of pre-existing cracks. In the first cycle after the peak stress, the specimen still showed strong load-carrying capacity. Subsequently, the bearing capacity gradually decreases and tends to a relatively stable residual stress.

#### AE hits and AE events

**Figure 8:**
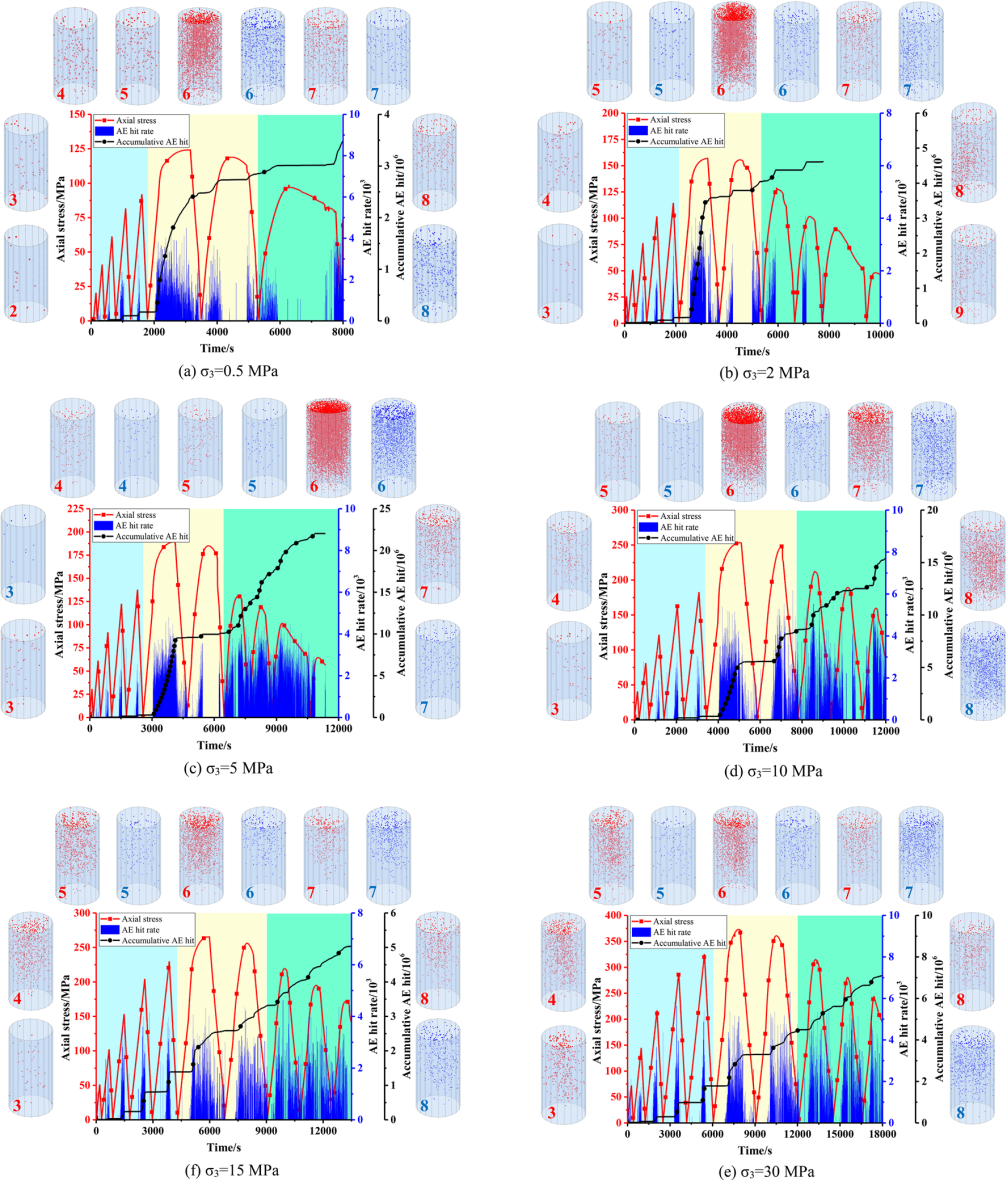
AE characteristics of Beishan granite under different confining pressures during CYC tests.

Stress, accumulative AE hits and AE hit rate versus time under different confining pressures are shown in Fig. 8. The distribution of AE events generated in each cycle is also shown in the figure. The red points represent AE events generated during loading, and the blue point represents AE events generated during unloading in each cycle.

In the first five cycles, it can be seen from the stress versus time curve that the rock specimen in this stage is mainly characterized by elastic deformation. The captured AE signals are mainly in the loading phase in each cycle. It mainly caused by micro creak closure, and extension of pre-existing micro cracks may contribute to it^26^. And in the unloading phase, there is no AE signal or only a small amount of AE signals. In addition, with the increase of confining pressure, the AE signal generated at this stage increases gradually.

The sixth and seventh cycles can be considered as the failure stage of the specimen. In this stage, AE signals are continuously generated at high frequencies due to crack coalescence, which leads to the generation of macro crack. And in the unloading phase of these two cycles, a considerable number of AE signals are generated. During the following cycles, the specimen enters the residual stage, the AE signals increase dramatically. And it can be seen that the AE signal generated in the unloading stage is not less than the AE signal generated in the loading stage.

#### AE amplitude and frequency

**Figure 9:**
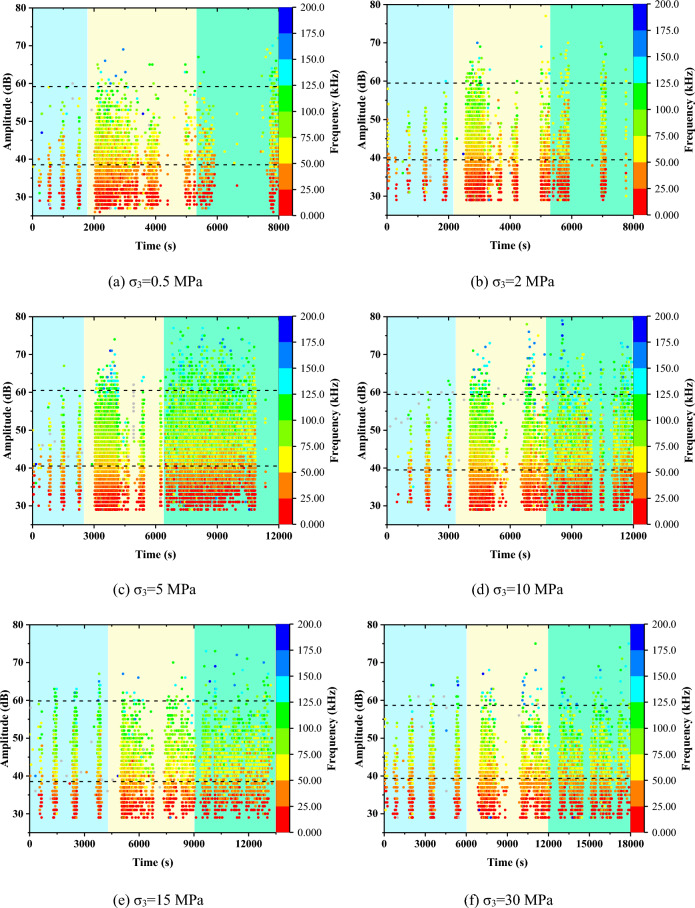
AE amplitude and frequency of Beishan granite under different confining pressures during CYC tests.

Figure 9 presents the dominant frequency-amplitude distributions of Beishan granite specimens in CYC tests. Its distribution range is the same as that of CTC test, as well as three frequency bands. In the elastic stage, with the increase of axial stress, the amplitude increases gradually. And signals are mainly concentrated in the low- and intermediate-frequency bands. In the damage and residual stage, the generated high-frequency signal is very few under relatively low confining pressure. Under relatively high confining pressure, more high-frequency signals are generated. But the number is still small.

#### AE *b*-value

**Figure 10:**
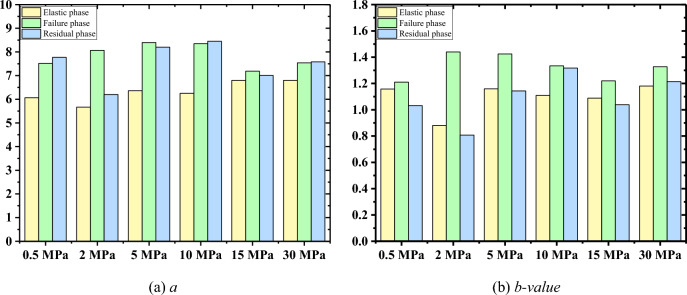
AE *a-*value and *b-value*s during the three loading stages of granite specimens during CYC tests.

According to the calculation method in Section “AE b-value”, AE *a*-values and *b*-values of the three stages in CYC experiment are calculated respectively, as shown in Fig. 10. As illustrated in Fig. 10, the smallest *a-*value is clearly obtained during elastic stage. While in the other two stages, the difference of *a-*value is small, except when the confining pressure is 2 MPa. This indicates that AE activity is weak in the elastic stage, and AE is more active in the failure and residual stages. The greatest *b-*value is obtained during failure stage, and the lowest value is mainly obtained during the residual stage. This demonstrates that micro cracks are thrived in the damage stage and in the residual stage the coalescence of micro cracks leads to the formation of macro cracks.

## Conclusion

In this paper, in order to study the damage characteristics of Beishan granite under single loading and multiple loading, CTC and CYC tests are carried out. And the following conclusions can be drawn.

During CTC test, the AE signals maintain at a very high level after dilatancy. But a decreasing trend of AE signals can be observed when the axial stress approaches the peak stress. Most of the AE waveforms have a dominant frequency of less than 200 kHz and amplitude of less than 80 dB. And AE signals can be divided into three frequency bands, low-frequency band, intermediate frequency band and high-frequency band. In addition, the obtained *b-value*s indicate that the relative proportion of micro- to macro-cracks increases first and then decreases with increasing confining pressure.

During CYC test, in the elastic stage, AE signals are mainly generated in the loading period. Also, we can see that the amplitude increases with the increase of loading stress. In the failure and residual stages, AE signals generated in the unloading period are considerable. The dominant frequency-amplitude distribution range is the same as that in single loading tests. The relatively high AE *a-value* and the greatest AE *b-value* confirms that micro cracks develop rapidly in the failure stage and lead to the formation of macro cracks in the residual stage.

In the future, more tests are needed to be conducted for further investigating the AE and fracturing characteristics of Beishan granite subjected to different conditions.

## Data Availability

The data used to support the findings of this study are available from the corresponding author upon request.
